# A phase 1, open-label, drug–drug interaction study of rucaparib with rosuvastatin and oral contraceptives in patients with advanced solid tumors

**DOI:** 10.1007/s00280-021-04338-7

**Published:** 2021-08-09

**Authors:** Mingxiang Liao, Krzysztof G. Jeziorski, Monika Tomaszewska-Kiecana, István Láng, Marek Jasiówka, Viera Skarbová, Piotr Centkowski, Rodryg Ramlau, Maria Górnaś, John Lee, Sarah Edwards, Jenn Habeck, Eileen Nash, Nikolay Grechko, Jim J. Xiao

**Affiliations:** 1 Clinical Pharmacology, Clovis Oncology, Inc 5500 Flatrion Pkwy, Boulder, CO 80301 USA; 2 Department of Gerontology, Public Health and Didactics, National Institute of Geriatrics, Rheumatology and Rehabilitation, Warsaw, Poland; 3 Maria Skłodowska-Curie National Research Institute of Oncology, Warsaw, Poland; 4 BioVirtus Research Site Sp. Z.O.O., BioVirtus Medical Centre, Józefów, Poland; 5 Oncology Unit, Istenhegy Private Health Center, Budapest, Hungary; 6 Gynecological Oncology Clinic, Centre of Oncology, Maria Skłodowska-Curie Memorial Institute, Krakow, Poland; 7 Pleiades Medical Centre, Krakow, Poland; 8 Department of Internal Medicine and Clinical Pharmacology, Summit Clinical Research, Bratislava, Slovakia; 9 Department of Oncology and Hematology, Provincial Specialist Hospital, Biala Podlaska, Poland; 10 Department of Oncology, Poznan University of Medical Sciences, Poznań, Poland; 11 Department of Chemotherapy, ATTIS Centre, Warsaw, Poland; 12 Regulatory Affairs, Clovis Oncology UK, Ltd., Cambridge, UK; 13 Medical Affairs, Clovis Oncology UK, Ltd., Cambridge, UK; 14 Biostatistics, Clovis Oncology, Inc., Boulder, CO USA; 15 Clinical Operations, Clovis Oncology, Inc., Boulder, CO USA; 16 Clinical Science, Clovis Oncology UK, Ltd., Cambridge, UK

**Keywords:** BCRP, Drug–drug interaction, Oncology, Oral contraceptives, Rucaparib

## Abstract

**Purpose:**

This study aimed at evaluating the effect of rucaparib on the pharmacokinetics of rosuvastatin and oral contraceptives in patients with advanced solid tumors and the safety of rucaparib with and without coadministration of rosuvastatin or oral contraceptives.

**Methods:**

Patients received single doses of oral rosuvastatin 20 mg (Arm A) or oral contraceptives ethinylestradiol 30 µg + levonorgestrel 150 µg (Arm B) on days 1 and 19 and continuous doses of rucaparib 600 mg BID from day 5 to 23. Serial blood samples were collected with and without rucaparib for pharmacokinetic analysis.

**Results:**

Thirty-six patients (*n* = 18 each arm) were enrolled and received at least 1 dose of study drug. In the drug–drug interaction analysis (*n* = 15 each arm), the geometric mean ratio (GMR) of maximum concentration (*C*_max_) with and without rucaparib was 1.29 for rosuvastatin, 1.09 for ethinylestradiol, and 1.19 for levonorgestrel. GMR of area under the concentration–time curve from time zero to last quantifiable measurement (AUC_0–last_) was 1.34 for rosuvastatin, 1.43 for ethinylestradiol, and 1.56 for levonorgestrel. There was no increase in frequency of treatment-emergent adverse events (TEAEs) when rucaparib was given with either of the probe drugs. In both arms, most TEAEs were mild in severity and considered unrelated to study treatment.

**Conclusion:**

Rucaparib 600 mg BID weakly increased the plasma exposure to rosuvastatin or oral contraceptives. Rucaparib safety profile when coadministered with rosuvastatin or oral contraceptives was consistent with that of rucaparib monotherapy. Dose adjustments of rosuvastatin and oral contraceptives are not necessary when coadministered with rucaparib.

**ClinicalTrials.gov** NCT03954366; **Date of registration** May 17, 2019.

**Supplementary Information:**

The online version contains supplementary material available at 10.1007/s00280-021-04338-7.

## Introduction

Rucaparib is a potent, oral, small-molecule inhibitor of poly(ADP-ribose) polymerase (PARP) 1, PARP2, and PARP3 [1]. In cancer cells with homologous recombination deficiency, synthetic lethality results when mutations in genes encoding proteins involved in the homologous recombination repair pathway are combined with rucaparib-induced inhibition of PARP proteins, leading to tumor cell death [1, 2]. Rucaparib is approved in the United States and the European Union as monotherapy for the maintenance treatment or treatment of adult patients with recurrent epithelial ovarian, fallopian tube, or primary peritoneal cancer [3, 4]. Recently, rucaparib also received accelerated approval from the US Food and Drug Administration as monotherapy for adult patients with a deleterious *BRCA1* or *BRCA2* mutation (germline and/or somatic)-associated metastatic castration-resistant prostate cancer [3].

The pharmacokinetic (PK) profile of rucaparib as a monotherapy was previously examined in patients from Study 10 (NCT01482715) [4, 5]. Among patients with advanced solid tumors, across all dosages (40–500 mg once daily [QD] or 240–840 mg twice daily [BID]), steady state of plasma rucaparib PK was achieved by day 8 [4]. Steady-state maximum concentration (*C*_max_) and area under the concentration–time curve (AUC) of rucaparib were approximately dose proportional with relatively short median time to *C*_max_ (*t*_max_; range 1.5–6.0 h).

In vitro study of the drug–drug interaction (DDI) potential of rucaparib found that rucaparib is an inhibitor of breast cancer resistance protein (BCRP) with a 50% inhibitory concentration (IC_50_) of 55 µM in transfected cells [6]. BCRP is an efflux transporter found on the apical membrane of intestinal epithelial cells, brain endothelial cells, and hepatocytes that can mediate the transport of drugs, such as rosuvastatin, sulfasalazine, and prazosin. It can play important roles in drug absorption, distribution, and elimination [7]. The inhibition of BCRP may significantly affect the efficacy and safety of its substrates in humans [8]. Additionally, rucaparib reversibly inhibited cytochrome P450 (CYP) enzymes, including CYP3As (IC_50_, 17.2–22.9 µM) in human liver microsomes, down-regulated CYP3A4 mRNA expression, and reduced CYP3A activities in human hepatocytes [6]. Following the in vitro study, a phase 1 study (NCT02740712) was conducted to evaluate the DDI of rucaparib and CYP enzyme substrates in patients with an advanced solid tumor [9]. At steady state, rucaparib weakly inhibited CYP3A [9]. These results suggest that steady-state rucaparib is likely to have a limited impact on the exposure of CYP3A substrates and would not reduce the clinical efficacy of oral contraceptives, which are often CYP3A substrates [10, 11]. However, there may still be unknown mechanisms of induction of CYP3A enzymes that could affect the PK and efficacy of oral contraceptives [12].

Taken together, the risk of a clinically relevant interaction between rucaparib and BCRP substrates or oral contraceptives cannot be excluded. Therefore, the current phase I study (NCT03954366) was conducted to specifically evaluate the DDIs between rucaparib and the BCRP substrate rosuvastatin (a 3-hydroxy-3-methylglutaryl coenzyme A [HMG Co-A] reductase inhibitor used for the treatment of hypercholesterolemia and for the prevention of major cardiovascular events [13]) and the common oral contraceptives, ethinylestradiol, and levonorgestrel. The primary objective of this study was to determine the effect of rucaparib on the PK of oral rosuvastatin and oral contraceptives. Secondary objectives were to characterize the steady-state PK of rucaparib and to determine the tolerability and safety of rucaparib with and without coadministration of rosuvastatin or oral contraceptives.

## Methods

### Patients

Male and female patients (Arm A) and female patients (Arm B) were eligible for enrollment if they were ≥ 18 years of age with a body mass index of 18.0 to 35.0 kg/m^2^, had histologically or cytologically confirmed advanced solid tumors with evidence of measurable disease per Response Evaluation Criteria In Solid Tumors version 1.1 [14], had Eastern Cooperative Oncology Group (ECOG) performance status of 0 or 1, and could potentially benefit from treatment with rucaparib in the opinion of the investigator. Patients must have had adequate bone marrow function (absolute neutrophil count ≥ 1.5 × 10^9^/L, platelets > 100 × 10^9^/L, hemoglobin ≥ 9 g/dL), liver function (aspartate aminotransferase [AST]/alanine aminotransferase [ALT] ≤ 3 × upper limit of normal [ULN], bilirubin ≤ 1.5 × ULN, serum albumin ≥ 30 g/L), and renal function (creatinine clearance [CrCL] ≥ 45 mL/min using the Cockcroft-Gault formula).

Key exclusion criteria for Arms A and B included anticancer treatment (chemotherapy, radiation, or other targeted agents) or experimental drugs of any kind within 14 days of starting treatment; unresolved grade ≥ 2 adverse events from prior therapies; pre-existing duodenal stent and/or any gastrointestinal disorder or defect that could interfere with absorption of rucaparib; or recent history of venous or arterial thromboembolic disease or major cardiac disease. Patients could not have consumed alcohol within 72 h or consumed any grapefruit products or products containing star fruit, Seville orange, pomegranate, pomelo, or their juices within 7 days of starting treatment or during the study.

Patients were also excluded from Arm A if they had ongoing use of rosuvastatin; use of another statin was also prohibited within 7 days prior to day 1 and during the study. In addition, patients were excluded if they had hypersensitivity to rosuvastatin or to any of the excipients; had current or history of clinically significant myopathy; or used inhibitors or inducers of BCRP, organic anion transporting polypeptides (OATP)1B1/OATP1B3, CYP2C9, or CYP2C19 within 14 days prior to day 1 and during the study. Patients were excluded from Arm B if they had ongoing use of any contraceptive drugs or previously had contraceptive implants or depot injections which may still be clinically effective; had history of hypersensitivity to ethinylestradiol or levonorgestrel or to any of the excipients; or used inhibitors or inducers of CYP3A4, CYP2C9, UDP-glucuronosyltransferase (UGT)1A1, or sulfotransferase (SULT)1E1 within 14 days prior to day 1 and during the study.

### Study design

**Fig. 1:**
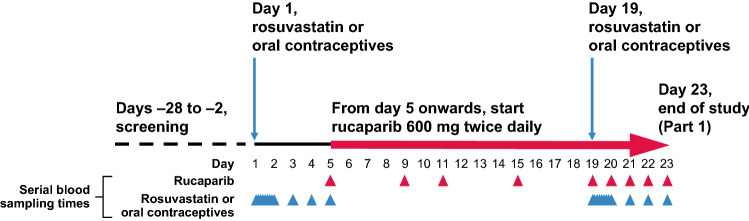
Study design

This was a 2-part, phase 1, open-label, 2-arm DDI study in patients with advanced solid tumors. In Part 1 of the study, patients were screened for eligibility from day − 28 to − 2 and visited the study site from day − 1 to 23 (Fig. 1). Male and female patients in Arm A received single oral doses of rosuvastatin 20 mg on days 1 and 19 and continuous oral doses of rucaparib 600 mg BID from day 5 to 23. Female patients in Arm B received single doses of the combined oral contraceptive pills (ethinylestradiol 30 µg + levonorgestrel 150 µg) on days 1 and 19 and continuous oral doses of rucaparib 600 mg BID from day 5 to 23. The PK of rosuvastatin (Arm A) and oral contraceptives (Arm B) without and with rucaparib was monitored on days 1 and 19, respectively. Serial blood samples were collected prior to the dosing of the probe drugs (rosuvastatin or oral contraceptives) and at 0.5, 1, 1.5, 2, 4, 6, 8, 24, 48, 72, and 96 h postdose on days 1 and 19. To check steady-state plasma concentrations for rucaparib, a blood sample was collected prior to the dosing of rucaparib on days 5, 9, 11, 15, and 19–23.

The safety population included patients who received at least 1 dose of study drug. The PK analysis population included patients who received the protocol-defined treatment, finished all or part of the PK assessments, and had sufficient PK data to calculate at least 1 primary PK parameter of the probe drug with or without rucaparib. The DDI analysis population included patients who completed the DDI assessment per protocol and had sufficient PK data to calculate *C*_max_ and AUC of the probe drug with and without rucaparib.

Results from Part 1 of the study are presented. After completion of Part 1, patients had the option to enter Part 2 of the study and continue rucaparib treatment until progression of disease, unacceptable toxicity, or discontinuation for other reasons based on investigator discretion and patient consent.

The study protocol was approved by the Independent Ethics Committee of each institution. The study was conducted in compliance with the International Council for Harmonisation Guideline for Good Clinical Practice, Food and Drug Administration regulatory requirements, and the Declaration of Helsinki. All patients signed informed consent forms prior to entering the study.

### Genotyping

The ATP binding cassette subfamily G member 2 (*ABCG2*) gene encodes BCRP, and the solute carrier organic anion transporter family member 1B1 (*SLCO1B1*) gene encodes OATP1B1. Because polymorphisms in both genes are associated with higher exposure to rosuvastatin [15, 16], the 3 polymorphisms *ABCG2* C421A, *SLCO1B1* T521C, and *SLCO1B1* A388G were analyzed for all patients in Arm A at Eurofins (Ebersberg, Germany). If a patient had homozygous *ABCG2* C421A, he or she was excluded from the DDI assessment of rosuvastatin. Patients who had homozygous and/or heterozygous *SLCO1B1* T521C or *SLCO1B1* A388G or heterozygous *ABCG2* C421A were not excluded.

### PK variables

The primary PK parameters calculated for rosuvastatin and oral contraceptives with and without rucaparib were *C*_max_, AUC from time zero to last quantifiable measurement (AUC_0–last_), and AUC from time zero to infinity (AUC_0–inf_). Other PK parameters, calculated for the probe drugs, included half-life (*t*_1/2_), *t*_max_, apparent total clearance of drug after oral administration (CL/F), and apparent volume of distribution during terminal phase (*V*_Z_/F). The PK parameter calculated for rucaparib at steady state was trough plasma concentration (*C*_min_). PK parameters were calculated by noncompartmental analysis using Phoenix WinNonlin, version 8.1 (Certara, Princeton, NJ).

The effect of rucaparib on the PK of oral rosuvastatin and oral contraceptives was determined based on the geometric mean ratio (GMR) of *C*_max_ and/or AUC of each probe drug with and without rucaparib.

### Plasma sample analysis

The plasma concentrations of rucaparib and rosuvastatin were measured by Q Squared Solutions BioSciences (Ithaca, NY) and oral contraceptives by PRA Bioanalytical Laboratory (Assen, The Netherlands) using validated high-performance liquid chromatography coupled in-line with tandem mass spectrometric detection methods (LC–MS/MS). Details are provided in Online Resource 1.

### Safety

Safety was assessed separately during the 3 treatment periods: before rucaparib dosing (day 1 to 5), rucaparib dosing alone (day 6 to 18), and rucaparib with and after probe drug (day 19 to 23). Safety and tolerability assessments were made based on adverse events (AEs), clinical laboratory results, vital signs, 12-lead electrocardiogram (ECG) measurements, body weight, physical examination, concomitant medication/procedures, and ECOG performance status. AEs were classified according to the Medical Dictionary for Regulatory Activities (MedDRA) classification system version 22.1 [17] and graded according to the Common Terminology Criteria for Adverse Events (CTCAE) version 4.03 [18].

### Statistical analyses

The effect of rucaparib on the PK parameters of rosuvastatin and oral contraceptives was evaluated by comparing the GMR and 90% confidence interval (CI) of the primary PK parameters (*C*_max_, AUC_0–last_, and AUC_0–inf_) for probe drugs with versus without coadministration of rucaparib. With the assumption that intrapatient PK variability would be 35% for the probe drugs, a total enrollment of 32 patients was planned for this study: 16 for Arm A and 16 for Arm B. The sample sizes were determined sufficient for the point estimate of the GMR of the probe drugs with versus without rucaparib to fall within the 90% CI of 0.82–1.23.

The primary PK parameters were natural log-transformed before assessment with a paired *t* test. Point estimates for the differences in means and corresponding 90% CI between 2 treatments (with or without rucaparib) for each probe drug were obtained and exponentiated to obtain GMs, GMRs, and respective 90% CI on the original scale.

Plasma concentrations and PK parameters for rosuvastatin and oral contraceptives with and without rucaparib were summarized with descriptive statistics, including number of patients, arithmetic mean, standard deviation (SD), coefficient of variation in percent (%CV), minimum, maximum, and GM with 90% CI.

## Results

### Patient demographics and genotyping

In total, 36 patients (Arm A, *n* = 18 and Arm B, *n* = 18) were enrolled in this study and received at least 1 dose of study drug. All 36 patients were included in the safety population. The median age of the enrolled patients was 63 years (range, 39–79 years) with a mean body mass index of 26.2 kg/m^2^ (Table 1). All patients were white and had received prior systemic anticancer therapy. Cancers of the lower digestive tract were the most common cancer type (27.8%).

**Table 1:** Summary of patient demographics and baseline characteristics

Characteristic	Arm A (*n* = 18)	Arm B (*n* = 18)	Overall (*N* = 36)
Age, median (range), y	61 (39–79)	64 (40–72)	63 (39–79)
Sex, *n* (%)			
Male	10 (55.6)	0	10 (27.8)
Female	8 (44.4)	18 (100.0)	26 (72.2)
Race, *n* (%)			
White	18 (100.0)	18 (100.0)	36 (100.0)
ECOG PS, *n* (%)			
0	7 (38.9)	10 (55.6)	17 (47.2)
1	11 (61.1)	8 (44.4)	19 (52.8)
BMI, mean (SD), kg/m^2^	26.9 (3.9)	25.5 (3.8)	26.2 (3.9)
Advanced solid tumor type, *n* (%)			
Large intestine cancer	7 (38.9)^a^	3 (16.7)^b^	10 (27.8)
Lung cancer	2 (11.1)	0	2 (5.6)
Prostate cancer	2 (11.1)	0	2 (5.6)
Ovarian cancer	2 (11.1)	7 (38.9)	9 (25.0)
Breast cancer	1 (5.6)	4 (22.2)	5 (13.9)
Uterine cancer	1 (5.6)	1 (5.6)	2 (5.6)
Pancreatic cancer	0	3 (16.7)	3 (8.3)
Other	3 (16.7)^c^	0	3 (8.3)
Prior therapy, *n* (%)			
Systemic anticancer therapy	18 (100.0)	18 (100.0)	36 (100.0)
Anticancer surgery	12 (66.7)	15 (83.3)	27 (75.0)
Anticancer radiotherapy	7 (38.9)	7 (38.9)	14 (38.9)

*BMI* body mass index, *N* total number of patients, *n* number of assessed patients, *SD* standard deviation

^a^Includes colon and rectal cancer

^b^Includes colon and anal cancer

^c^Includes 1 patient each with renal cancer, cervical cancer, or papilla vateri carcinoma

Sixteen patients from Arm A who completed all the assessments in Part 1 were genotyped for *ABCG2* and *SLCO1B1* variants. Five patients carried the *ABCG2* C421A mutation, 4 of whom had a heterozygous and 1 had a homozygous mutation. The patient with homozygous *ABCG2* C421A mutation was included in the PK analysis population but was excluded from the DDI analysis. A total of 12 patients had *SLCO1B1* mutations: 4 had a homozygous *SLCO1B1* A388G mutation, 1 had a homozygous *SLCO1B1* T521C mutation and a heterozygous *SLCO1B1* A388G mutation, and 7 had heterozygous *SLCO1B1* A388G and/or T521C mutations.

### Study populations

In Arm A, 3 patients were excluded from the DDI analysis population: 2 patients did not complete Part 1 of the study, and 1 had a homozygous *ABCG2* C421A mutation (as described above). One of the 2 patients who did not complete the study was also excluded from the PK analysis population because vomiting occurred within 4 h postdose on day 1. In Arm B, 3 patients were excluded from the DDI analysis population: 2 did not complete Part 1 of the study, and 1 had an irregular PK profile. Overall, 35 patients were included in the PK analysis population (Arm A, *n* = 17 and Arm B, *n* = 18), and 30 patients were included in the DDI analysis population (Arm A, *n* = 15 and Arm B, *n* = 15).

### Pharmacokinetics and drug–drug interactions

Predose blood sampling found that the arithmetic mean *C*_min_ values of rucaparib were similar from day 9 to 23, suggesting that the steady-state PK of rucaparib was achieved by day 9 (4 days of continuous rucaparib dosing from day 5) (Online Resource 2). The steady-state plasma concentrations of rucaparib were comparable to historical data [4].

**Fig. 2:**
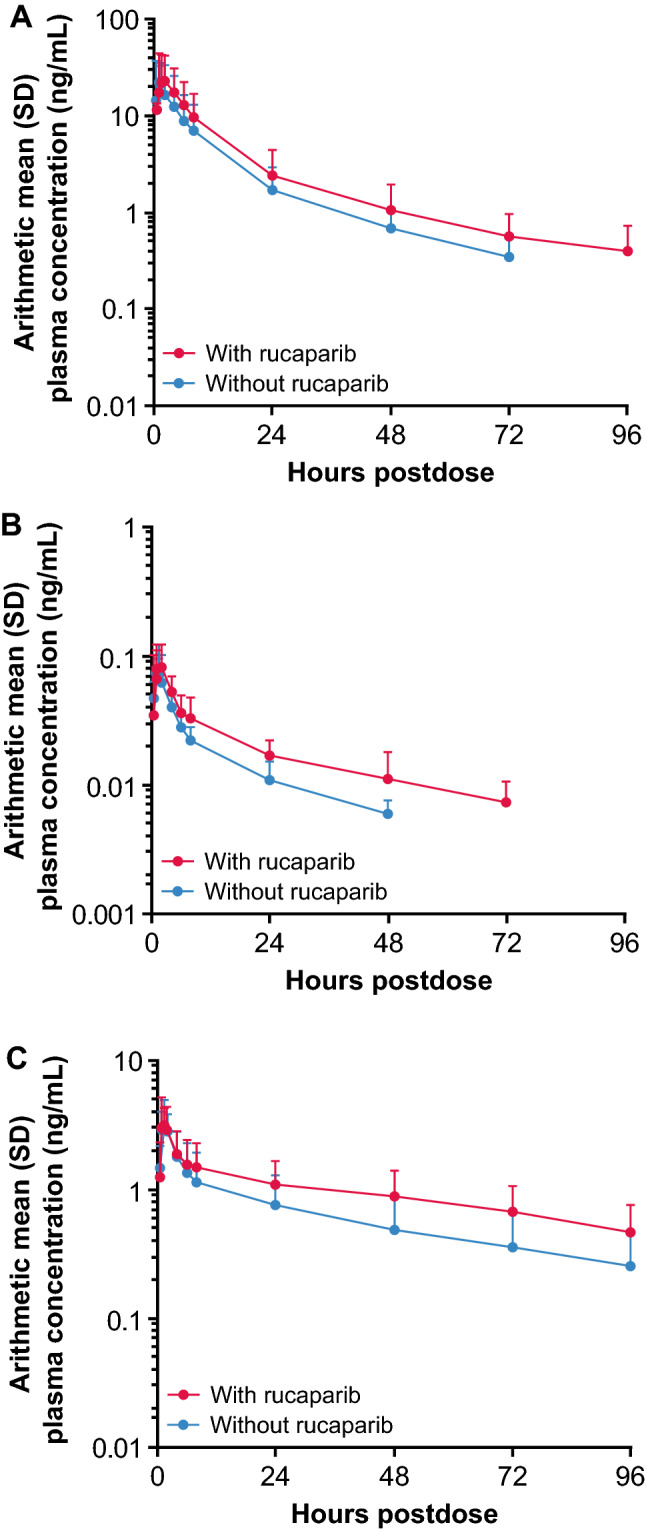
Arithmetic mean (SD) plasma concentration–time profiles for rosuvastatin (**A**) and oral contraceptives, ethinylestradiol (**B**) and levonorgestrel (**C**), with and without rucaparib (PK analysis population). *PK* pharmacokinetics, *SD* standard deviation

**Fig. 3:**
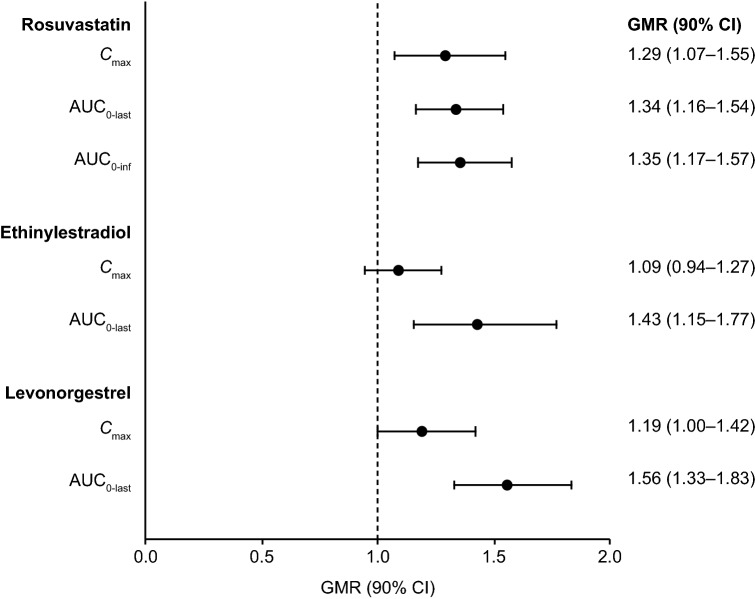
The effect of rucaparib on the PK of rosuvastatin and oral contraceptives (DDI analysis population). *AUC* area under the concentration–time curve, *AUC*_*0–inf*_ AUC extrapolated from time 0 to infinity, *AUC*_*0–last*_ AUC from time 0 up to the last time point with a quantifiable concentration, *CI* confidence interval, *C*_max_ maximum plasma concentration, *DDI* drug–drug interaction, *GMR* geometric mean ratio

The arithmetic mean plasma concentration–time profiles for rosuvastatin and each of the oral contraceptives with and without rucaparib are shown in Fig. 2. PK parameters of rosuvastatin and oral contraceptives with and without rucaparib are summarized in Table 2. The median *t*_max_ values of the probe drugs with and without rucaparib were similar and ranged from 1.0 to 2.0 h; the median *t*_max_ of rosuvastatin and ethinylestradiol increased by 0.5 h with rucaparib, but the median *t*_max_ of levonorgestrel remained the same with and without rucaparib. For rosuvastatin, the GM values for *C*_max_, AUC_0–last_, and AUC_0–inf_ were slightly higher with rucaparib than without rucaparib, and GM values of CL/F and *V*_Z_/F were lower with rucaparib than without rucaparib. Rucaparib weakly increased *C*_max_ (GMR, 1.29), AUC_0–last_ (GMR, 1.34), and AUC_0–inf_ (GMR, 1.35) of rosuvastatin (Fig. 3). For the oral contraceptives, the GM values for *C*_max_ were similar, AUC_0–last_ and AUC_0–inf_ were higher with rucaparib than without rucaparib, and CL/F and *V*_Z_/F were lower with rucaparib than without rucaparib. For both ethinylestradiol and levonorgestrel, rucaparib marginally increased *C*_max_ (GMR, 1.09 and 1.19, respectively) and weakly increased AUC_0–last_ (GMR, 1.43 and 1.56, respectively).

**Table 2:** Summary of PK parameters of rosuvastatin and oral contraceptives with and without rucaparib (PK analysis population)

PK parameter	Rosuvastatin		Ethinylestradiol		Levonorgestrel	
	Without rucaparib (*n* = 17)	With rucaparib (*n* = 18)	Without rucaparib (*n* = 18)	With rucaparib (*n* = 18)	Without rucaparib (*n* = 18)	With rucaparib (*n* = 18)
*C*_max_, ng/mL						
*n*	17	16	18	17	18	17
Mean (SD)	20.5 (25.4)	25.4 (21.0)	0.0792 (0.0311)	0.0893 (0.0443)	3.32 (1.13)	3.77 (1.70)
GM (%CV)	13.0 (116)	18.1 (107)	0.0732 (44.3)	0.0784 (59.7)	3.17 (30.7)	3.43 (47.3)
Median (range)	10.2 (2.6–106)	18.1 (5.1–72.7)	0.0734 (0.0255–0.151)	0.0756 (0.025–0.167)	3.29 (1.88–7.08)	3.48 (1.46–7.23)
AUC_0–last_, h × ng/mL						
*n*	17	16	18	17	18	17
Mean (SD)	193 (156)	266 (209)	0.804 (0.370)	1.24 (0.484)	59.7 (35.3)	86 (39.9)
GM (%CV)	145 (95.9)	200 (95.9)	0.714 (57.4)	1.15 (43.8)	52.9 (49.6)	77.5 (51.5)
Median (range)	141 (21.9–552)	208 (42.7–850)	0.775 (0.231–1.54)	1.18 (0.486–2.15)	45.3 (30.3–149)	80.4 (28.1–182)
AUC_0–inf_, h × ng/mL						
*n*	16	16	11	12	10	7
Mean (SD)	192 (158)	276 (217)	0.994 (0.257)	1.49 (0.505)	72.7 (43.9)	109 (49.9)
GM (%CV)	145 (94.0)	210 (93.0)	0.962 (28.4)	1.41 (35.1)	64.0 (53.0)	102 (40.6)
Median (range)	145 (23.0–559)	212 (46.1–894)	1.00 (0.537–1.37)	1.37 (0.806–2.37)	55.4 (39.1–168)	87.3 (62.7–214)
*t*_max_, h						
*n*	17	16	18	17	18	17
Median (range)	1.5 (0.50–4.00)	2.0 (0.50–6.00)	1.00 (0.50–2.00)	1.50 (1.00–47.5)	1.51 (0.97–4.00)	1.50 (1.00–47.5)
*t*_1/2_, h						
*n*	16	16	16	16	16	15
Mean (SD)	20.6 (13.1)	18.5 (8.49)	17.0 (5.69)	30.0 (25.7)	41.1 (15.9)	50.0 (19.7)
GM (%CV)	17.5 (64.4)	16.6 (51.8)	15.9 (40.9)	24.8 (63.4)	38.5 (38.5)	46.6 (40.4)
Median (range)	17.6 (5.17–60.5)	16.1 (6.25–32.8)	18.1 (7.29–25.1)	27.1 (9.27–122)	37.1 (22.4–75.7)	43.6 (23.6–84.5)
CL/F, L/h						
*N*	16	16	11	12	10	7
Mean (SD)	191 (201)	128 (109)	32.4 (9.82)	22.4 (7.54)	2.57 (1.01)	1.57 (0.531)
GM (%CV)	138 (94.0)	95.3 (93.0)	31.2 (28.4)	21.2 (35.1)	2.34 (53.0)	1.48 (40.6)
Median (range)	138 (35.8–870)	94.3 (22.4–434)	30.0 (21.8–55.8)	21.8 (12.7–37.2)	2.71 (0.891–3.84)	1.72 (0.701–2.39)
*V*_Z_/F, L						
*n*	16	16	11	12	10	7
Mean (SD)	4620 (3730)	2960 (2250)	792 (175)	684 (217)	113 (43.9)	82.8 (30.0)
GM (%CV)	3490 (94.4)	2280 (85.0)	772 (24.9)	653 (32.7)	103 (55.6)	77.6 (42.7)
Median (range)	3730 (792–15,200)	1840 (815–8100)	772 (418–1140)	620 (385–1000)	114 (30.6–188)	85.6 (35.9–132)

If percentage extrapolation was > 20% or *R*_sq_ was ≤ 0.80, then AUC_0–inf_, CL/F, and *V*_Z_/F were excluded from summary statistics. If *R*_sq_ was ≤ 0.80, then *t*_1/2_ was excluded from summary statistics

*%CV* coefficient of variation in percent, *AUC* area under the concentration–time curve, *AUC*_*0–inf*_ AUC extrapolated from time 0 to infinity, *AUC*_*0–last*_ AUC from time 0 up to the last time point with a quantifiable concentration, *CL/F* apparent total clearance of drug after oral administration, *C*_max_ maximum plasma concentration, *GM* geometric mean, *h* hours, *N* total number of patients, *n* number of assessed patients, *PK* pharmacokinetics, *R*_*sq*_ R-squared, *SD* standard deviation, *t*_1/2_ half-life, *t*_max_ median time to maximum concentration, *V*_Z_*/F* apparent volume of distribution during terminal phase

### Safety

In Arm A, 1 (1/18; 5.6%) patient experienced grade 1 diarrhea, and 1 patient had grade 1 vomiting and discontinued Part 1 on day 1 during treatment with rosuvastatin prior to rucaparib dosing (Online Resource 3). Of 17 patients who received rucaparib alone from day 5 to 19, 10 (58.8%) experienced at least 1 TEAE, 3 of which were considered related to rucaparib by the investigator (grade 2 vomiting, grade 1 abdominal distension, and grade 1 increased blood creatinine). After day 19, 4 (4/16; 25.0%) patients who received rucaparib and a second dose of rosuvastatin experienced TEAEs, including grade 1 vaginal hemorrhage, grade 2 dyspepsia, grade 2 urinary tract infection, grade 2 increased body temperature, and grade 2 prolonged electrocardiogram QT (1/16 each; 6.3%); none were considered treatment-related.

In Arm B, 2 (2/18; 11.1%) patients experienced TEAEs (grade 1 diarrhea and grade 2 vomiting) during treatment with oral contraceptives prior to rucaparib dosing (Online Resource 3). Of 18 patients who received rucaparib alone from day 5 to 19, 9 (9/18; 50.0%) patients reported at least 1 TEAE during treatment. After day 19, 6 patients (6/17; 35.3%) administered with both rucaparib and oral contraceptives experienced grade 2 anemia, grade 2 thrombocytopenia, grade 1 abdominal pain, grade 1 nausea, grade 1 vomiting, grade 1 erythema, grade 2 increased alanine aminotransferase, and grade 3 cerebrovascular accident (1/17 each; 5.9%). Overall, 7 patients experienced TEAEs that were considered related to rucaparib, and 3 patients experienced TEAEs that were considered related to treatment (rucaparib and oral contraceptives) by the investigator. The most common rucaparib- and treatment-related TEAEs were grade 1 dysgeusia and grade 1 vomiting.

Three patients withdrew from Part 1 of the study due to TEAEs: 1 patient in Arm A due to grade 3 deep vein thrombosis, grade 4 pulmonary embolism, and grade 2 sinus tachycardia; 1 patient in Arm B due to grade 3 acute kidney injury; and 1 patient in Arm B due to a grade 3 cerebrovascular accident, which was a serious adverse event; all were considered unrelated to treatment. The patient in Arm A who withdrew from Part 1 was transitioned to receive rucaparib in Part 2 of the study. One death occurred in Arm B due to disease progression.

Clinically relevant ECG findings were captured from 2 patients in Arm A: 1 patient had grade 1 and grade 2 clinically significant prolongation of QTc intervals on days 18 and 23, respectively; another patient had grade 2 sinus tachycardia on day 18. Both patients fully recovered by the end of the study, and neither event was considered related to treatment with rucaparib. There were no other clinically relevant treatment-related trends observed with respect to clinical laboratory, vital signs, ECOG performance status, or physical examination.

## Discussion

Based on prior studies indicating inhibition of BCRP and CYP3A by rucaparib [6, 9] and the possibility of having unknown mechanisms of induction of CYP3As that could impact the PK of oral contraceptives [12], the present study evaluated the effect of rucaparib on the PK of the BRCP substrate rosuvastatin and the oral contraceptives ethinylestradiol and levonorgestrel in patients with advanced solid tumors, as well as the safety of rucaparib with and without coadministration of the probe drugs.

Among 32 patients (Arm A, *n* = 16; Arm B, *n* = 16) who completed the study, 2 patients (1 patient per arm) were not evaluable for the DDI assessment due to presence of homozygous *ABCG2* C421A mutation or an irregular PK profile. However, the PK analysis of the remaining 30 patients (15 patients per arm) allowed precise estimation of the GMRs for PK parameters with narrow 90% CIs.

To allow a full induction of CYP3As (if any), at least 7 days of CYP inducer dosing at steady-state were required before the administration of a probe drug [19]. In the current study, apparent steady-state of rucaparib was achieved following 4 days of dosing (on day 9), indicating that the plasma concentrations of rucaparib were sufficient to evaluate the interactions between rucaparib and oral contraceptives (on day 19). The rucaparib arithmetic mean *C*_min_ were similar from day 9 to 23 and consistent with historic data [4, 5], suggesting that the coadministration of rosuvastatin and oral contraceptives had no effect on rucaparib PK.

In Arm A, large individual differences in *C*_max_ and AUC of rosuvastatin were observed in patients with cancer. The complex disease state [20] and confounding factors (eg, hepatic impairment, genetic polymorphisms of CYPs and transporters) in patients in this study have likely contributed to PK variability of rosuvastatin. However, the effect of high between-subject PK variability on DDI assessment was largely mitigated by the study design of sequential dosing of rosuvastatin and oral contraceptives without and with rucaparib.

As expected, given the prior evidence that rucaparib inhibits BCRP [6], steady-state rucaparib 600 mg BID weakly increased *C*_max_ and AUC for rosuvastatin up to 1.29- to 1.35-fold. The similar GMRs for *C*_max_ and AUC suggested rucaparib increased rosuvastatin exposure mainly through the inhibition of BCRP in the gut to improve the oral absorption of rosuvastatin. Rosuvastatin dose adjustments are recommended when coadministration with another drug results in a twofold or higher increase in rosuvastatin exposure [21]. Therefore, no dose adjustment is recommended when rosuvastatin is coadministered with rucaparib. Nevertheless, caution should be used when extrapolating the results to other BCRP substrates.

The genetic polymorphisms *SLCO1B1* A388G, *SLCO1B1* T521C, and *ABCG2* C421A could significantly affect rosuvastatin PK [22]. According to a published report, 10.7% of Caucasians have a heterozygous *ABCG2* C421A mutation and 2.5% have a homozygous *ABCG2* C421A mutation [23]. In comparison, the allele frequencies among 16 patients in Arm A of this study were slightly higher with 4 patients having a heterozygous *ABCG2* C421A mutation (25%) and 1 having a homozygous *ABCG2* C421A mutation (6.3%). In an additional analysis, rosuvastatin exposure GMRs (*C*_max_ and AUC) for the patient with a homozygous *ABCG2* C421A mutation were approximately 2.1- to 3.3-fold in the presence and absence of rucaparib, which were greater than exposure GMRs (approximately 1.3- to 1.4-fold) for the 15 patients without a homozygous *ABCG2* C421A mutation in Arm A. In contrast, the mean exposure GMRs for 5 patients with a homozygous *SLCO1B1* mutation were consistent with the GMRs for 11 patients without a homozygous *SLCO1B1* mutation in our study. Given the small sample size (*n* = 1 or *n* = 5), the impact of *ABCG2* or *SLCO1B1* homozygous mutations on rosuvastatin DDI assessment remains inconclusive.

Per the European Medicines Agency’s guideline on the investigation of drug interactions, a clinical DDI study of oral contraceptives should be considered if the investigational drug (ie, rucaparib) is a CYP3A inhibitor or inducer, and/or also inhibits other enzymes that metabolize ethinylestradiol (ie, CYP2C9) [24]. In the prior phase 1 study, rucaparib inhibited both CYP3A and CYP2C9 in patients with an advanced solid tumor, but the inhibitions were weak (AUC_0–inf_ GMR of 1.38 and AUC_0–96 h_ GMR of 1.49) [9]. In this study, the PK data in Arm B demonstrated that the *C*_max_ for ethinylestradiol and levonorgestrel marginally increased (< 1.25-fold) with rucaparib, but the AUC increased up to approximately 1.4- to 1.6-fold for ethinylestradiol and levonorgestrel, respectively, in the presence of steady-state rucaparib plasma concentrations. These results suggest that rucaparib increased the exposure to oral contraceptives mostly through inhibiting their elimination. There were also 2 patients in Arm B who were taking atorvastatin, which, like other statins, has been reported to weakly increase exposure to oral contraceptives [25]. Although the oral contraceptive exposure ratios of these 2 patients were consistent with the exposure ratios of other patients who were not on statin therapy (data not shown), the effect of atorvastatin may not have been apparent as these patients were on stable doses of atorvastatin throughout the study. Overall, as the interaction between rucaparib and ethinylestradiol and levonorgestrel resulted in mild increases to ethinylestradiol and levonorgestrel AUC, it is unlikely that coadministration would reduce the efficacy of oral contraceptives. Furthermore, changing levels of contraceptive hormones would not necessarily translate into increased toxicity, as hormone levels vary widely within and between individuals [26]. Therefore, no dose adjustment is recommended when oral contraceptives are coadministered with rucaparib.

When the validated LC–MS/MS method was used to quantify the oral contraceptives in human plasma samples, there was a bioanalytical challenge associated with the low dose of ethinylestradiol (30 µg): ethinylestradiol was detected in the human plasma up to 48 h (without rucaparib) or 72 h (with rucaparib) postdose, and the ethinylestradiol AUC_0–inf_ was not accurately determined due to the high (> 20%) percentage of extrapolation in 7 out of 18 patients when oral contraceptives were dosed alone and 5 out of 17 patients when oral contraceptives were dosed with rucaparib. As a consequence, AUC_0–inf_ was excluded from the DDI assessment of oral contraceptives.

Prior studies have shown that the dose of rosuvastatin should be reduced (to 10 mg QD or less) when coadministered with BCRP inhibitors (eg, atazanavir, lopinavir, and ritonavir) [27] to avoid DDIs and an increased risk of myopathy [13]. In this study, the majority of TEAEs reported were mild in severity (grade 1 or 2). The most frequently reported TEAE of any grade was diarrhea in both arms of the study. None of patients reported myopathy. Although the overall safety and tolerability of rucaparib with coadministration of rosuvastatin or oral contraceptives were consistent with other clinical studies with rucaparib monotherapy [5, 9, 28–30], the safety data are limited because of the small patient population. Moreover, patients received only single oral doses of the probe drugs with coadministration of the probe drugs and rucaparib (occurring only on day 19) rather than continuously.

In conclusion, results from this study suggest rucaparib weakly increased the exposure to rosuvastatin, ethinylestradiol, and levonorgestrel. The limited impact on the probe drug exposures suggest dose adjustments of rosuvastatin and oral contraceptives are not necessary when coadministered with rucaparib.

## Supplementary Information

Below is the link to the electronic supplementary material.

Supplementary file 1 (DOCX 81 kb)

## Data Availability

Requests for de-identified datasets for the results reported in this publication will be made available to qualified researchers following submission of a methodologically sound proposal to medinfo@clovisoncology.com. Data will be made available for such requests following online publication of this article and for 1 year thereafter in compliance with applicable privacy laws, data protection, and requirements for consent and anonymization. Data will be provided by Clovis Oncology, Inc.

## References

[1] Robillard L, Nguyen M, Harding TC, Simmons AD (2017). In vitro and in vivo assessment of the mechanism of action of the PARP inhibitor rucaparib. Cancer Res.

[2] Drew Y, Mulligan EA, Vong WT, Thomas HD, Kahn S, Kyle S, Mukhopadhyay A, Los G, Hostomsky Z, Plummer ER, Edmondson RJ, Curtin NJ (2011). Therapeutic potential of poly(ADP-ribose) polymerase inhibitor AG014699 in human cancers with mutated or methylated *BRCA1* or *BRCA2*. J Natl Cancer Inst.

[3] Rubraca (rucaparib) tablets [prescribing information] (2020). Clovis Oncology, Inc., Boulder

[4] Shapiro GI, Kristeleit R, Burris HA, LoRusso P, Patel MR, Drew Y, Giordano H, Maloney L, Watkins S, Goble S, Jaw-Tsai S, Xiao J (2019). Pharmacokinetic study of rucaparib in patients with advanced solid tumors. Clin Pharmacol Drug Dev.

[5] Kristeleit R, Shapiro GI, Burris HA, Oza AM, LoRusso P, Patel MR, Domchek SM, Balmana J, Drew Y, Chen LM, Safra T, Montes A, Giordano H, Maloney L, Goble S, Isaacson J, Xiao J, Borrow J, Rolfe L, Shapira-Frommer R (2017). A phase I-II study of the oral PARP inhibitor rucaparib in patients with germline BRCA1/2-mutated ovarian carcinoma or other solid tumors. Clin Cancer Res.

[6] Liao M, Jaw-Tsai S, Beltman J, Simmons AD, Harding TC, Xiao JJ (2020). Evaluation of in vitro absorption, distribution, metabolism, and excretion and assessment of drug-drug interaction of rucaparib, an orally potent poly(ADP-ribose) polymerase inhibitor. Xenobiotica.

[7] Mao Q, Unadkat JD (2015). Role of the breast cancer resistance protein (BCRP/ABCG2) in drug transport–an update. AAPS J.

[8] Lee CA, O'Connor MA, Ritchie TK, Galetin A, Cook JA, Ragueneau-Majlessi I, Ellens H, Feng B, Taub ME, Paine MF, Polli JW, Ware JA, Zamek-Gliszczynski MJ (2015). Breast cancer resistance protein (ABCG2) in clinical pharmacokinetics and drug interactions: practical recommendations for clinical victim and perpetrator drug-drug interaction study design. Drug Metab Dispos.

[9] Xiao JJ, Nowak D, Ramlau R, Tomaszewska-Kiecana M, Wysocki PJ, Isaacson J, Beltman J, Nash E, Kaczanowski R, Arold G, Watkins S (2019). Evaluation of drug–drug interactions of rucaparib and CYP1A2, CYP2C9, CYP2C19, CYP3A, and P-gp substrates in patients with an advanced solid tumor. Clin Transl Sci.

[10] Yaz (drospirenone/ethinyl estradiol) tablets [prescribing information] (2012). Bayer HealthCare Pharmaceuticals Inc., Wayne

[11] Skyla (levonorgestrel-releasing intrauterine system) [prescribing information] (2017). Bayer HealthCare Pharmaceuticals Inc., Whippany

[12] Agency EM (2008) Guideline on risk assessment of Medicinal Products on Human Reproduction and Lactation: From Data to Labellingrrr. https://www.ema.europa.eu/en/documents/scientific-guideline/guideline-risk-assessment-medicinal-products-human-reproduction-lactation-data-labelling_en.pdf. Accessed 13 Nov 2020

[13] Crestor (rosuvastatin calcium) tablets [prescribing information] (2016). AstraZeneca Pharmaceuticals LP, Wilmington

[14] Eisenhauer EA, Therasse P, Bogaerts J, Schwartz LH, Sargent D, Ford R, Dancey J, Arbuck S, Gwyther S, Mooney M, Rubinstein L, Shankar L, Dodd L, Kaplan R, Lacombe D, Verweij J (2009). New response evaluation criteria in solid tumours: revised RECIST guideline (version 1.1). Eur J Cancer.

[15] Department of Health and Human Services, FDA, Center for Drug Evaluation and Research (CDER), Center for Biologics Evaluation and Research (CBER) Guidance for Industry. Drug-induced Liver Injury: Premarketing Clinical Evaluation (2009). https://www.fda.gov/regulatory-information/search-fda-guidance-documents/drug-induced-liver-injury-premarketing-clinical-evaluation. Accessed 7 Jun 2019

[16] Giacomini KM, Balimane PV, Cho SK, Eadon M, Edeki T, Hillgren KM, Huang SM, Sugiyama Y, Weitz D, Wen Y, Xia CQ, Yee SW, Zimdahl H, Niemi M, International Transporter Consortium (2013). International Transporter Consortium commentary on clinically important transporter polymorphisms. Clin Pharmacol Ther.

[17] Brown EG, Wood L, Wood S (1999). The medical dictionary for regulatory activities (MedDRA). Drug Saf.

[18] Guideline on the investigation of drug interactions. Committee for Human Medicinal Products. http://www.ema.europa.eu/ema/index.jsp?curl=pages/regulation/general/general_content_001277.jsp&mid=WC0b01ac0580032ec5. Accessed 30 Sept 2017

[19] Yang J, Liao M, Shou M, Jamei M, Yeo KR, Tucker GT, Rostami-Hodjegan A (2008). Cytochrome p450 turnover: regulation of synthesis and degradation, methods for determining rates, and implications for the prediction of drug interactions. Curr Drug Metab.

[20] Undevia SD, Gomez-Abuin G, Ratain MJ (2005). Pharmacokinetic variability of anticancer agents. Nat Rev Cancer.

[21] Crestor (rosuvastatin calcium) tablets [summary of product characteristics] (2020). Aurobindo Pharma - Milpharm Ltd., Ruislip

[22] Chu X, Liao M, Shen H, Yoshida K, Zur AA, Arya V, Galetin A, Giacomini KM, Hanna I, Kusuhara H, Lai Y, Rodrigues D, Sugiyama Y, Zamek-Gliszczynski MJ, Zhang L, International Transporter Consortium (2018). Clinical probes and endogenous biomarkers as substrates for transporter drug-drug interaction evaluation: perspectives from the International Transporter Consortium. Clin Pharmacol Ther.

[23] de Jong FA, Marsh S, Mathijssen RH, King C, Verweij J, Sparreboom A, McLeod HL (2004). ABCG2 pharmacogenetics: ethnic differences in allele frequency and assessment of influence on irinotecan disposition. Clin Cancer Res.

[24] European Medicines Agency (2012) Guideline on the investigation of drug interactions. https://www.ema.europa.eu/en/documents/scientific-guideline/guideline-investigation-drug-interactions-revision-1_en.pdf. Accessed 29 Jan 2021

[25] Gavronski M, Volmer D, Hartikainen S, Zharkovsky A (2015). Potential drug interactions with statins: Estonian register-based study. Open Med (Wars).

[26] Nanda K, Stuart GS, Robinson J, Gray AL, Tepper NK, Gaffield ME (2017). Drug interactions between hormonal contraceptives and antiretrovirals. AIDS.

[27] Bierman WF, Scheffer GL, Schoonderwoerd A, Jansen G, van Agtmael MA, Danner SA, Scheper RJ (2010). Protease inhibitors atazanavir, lopinavir and ritonavir are potent blockers, but poor substrates, of ABC transporters in a broad panel of ABC transporter-overexpressing cell lines. J Antimicrob Chemother.

[28] Coleman RL, Oza AM, Lorusso D, Aghajanian C, Oaknin A, Dean A, Colombo N, Weberpals JI, Clamp A, Scambia G, Leary A, Holloway RW, Gancedo MA, Fong PC, Goh JC, O'Malley DM, Armstrong DK, Garcia-Donas J, Swisher EM, Floquet A, Konecny GE, McNeish IA, Scott CL, Cameron T, Maloney L, Isaacson J, Goble S, Grace C, Harding TC, Raponi M, Sun J, Lin KK, Giordano H, Ledermann JA (2017). Rucaparib maintenance treatment for recurrent ovarian carcinoma after response to platinum therapy (ARIEL3): a randomised, double-blind, placebo-controlled, phase 3 trial. Lancet.

[29] Swisher EM, Lin KK, Oza AM, Scott CL, Giordano H, Sun J, Konecny GE, Coleman RL, Tinker AV, O'Malley DM, Kristeleit RS, Ma L, Bell-McGuinn KM, Brenton JD, Cragun JM, Oaknin A, Ray-Coquard I, Harrell MI, Mann E, Kaufmann SH, Floquet A, Leary A, Harding TC, Goble S, Maloney L, Isaacson J, Allen AR, Rolfe L, Yelensky R, Raponi M, McNeish IA (2017). Rucaparib in relapsed, platinum-sensitive high-grade ovarian carcinoma (ARIEL2 Part 1): an international, multicentre, open-label, phase 2 trial. Lancet Oncol.

[30] Drew Y, Ledermann J, Hall G, Rea D, Glasspool R, Highley MS, Jayson GC, Sludden J, Murray J, Jamieson D, Halford S, Acton G, Backholer Z, Mangano R, Boddy A, Curtin N, Plummer E (2016). Phase 2 multicentre trial investigating intermittent and continuous dosing schedules of the poly(ADP-ribose) polymerase inhibitor rucaparib in germline BRCA mutation carriers with advanced ovarian and breast cancer. Br J Cancer.

