# Supplementary material for: A phase 1, open-label, drug–drug interaction study of rucaparib with rosuvastatin and oral contraceptives in patients with advanced solid tumors
Source: Cancer Chemother Pharmacol. 2021 Aug 9;88(5):887–97. doi: 10.1007/s00280-021-04338-7 (PMC8484168; doi:10.1007/s00280-021-04338-7)
Supplement: Supplementary file 1 — (DOCX 81 kb) [file 280_2021_4338_MOESM1_ESM.docx]

# Online Resource Materials

## **Online Resource 1** Analytical methods

Plasma concentrations of rucaparib or rosuvastatin were determined by Q Squared Solutions BioSciences (Ithaca, NY; formerly Advion Bioanalytical Laboratories) using validated liquid chromatography-tandem mass spectrometry (LC-MS/MS) methods. Blood samples were collected in tubes containing dipotassium ethylenediaminetetraacetic acid.

For rosuvastatin, a 96-well protein precipitation extraction procedure was developed to isolate the analyte from plasma samples of 100 μL aliquots. The resulting samples were subject to LC-MS/MS analysis using a selected reaction monitoring (SRM) method with deuterium-labeled rosuvastatin (rosuvastatin-d_6_) as the internal standard (IS). The LC consisted of a Gemini NX C18 column (2 x 50 mm, 3 μm, Phenomenex, Torrance, CA) and was eluted with a gradient consisting of 0.1% acetic acid in water as mobile phase A and with 100% acetonitrile as mobile phase B at an initial flow rate of 0.5 mL/min at room temperature. The MS/MS consisted of an AB SCIEX API5000 system and was operated with Analyst version 1.6.2 in TurboIonSpray and positive ionization mode. Rosuvastatin and rosuvastatin-d_6_ were quantified by SRMs of m/z 483.2→259.1 and m/z 488.2→264.2, respectively. The concentration range for quantitation was 0.1–100 ng/mL. Plasma concentrations below the lower limit of quantitation (0.1 ng/mL) were treated as “0” when calculating summary statistics.

For rucaparib, a 96-well protein precipitation extraction procedure was developed to isolate the analyte from plasma samples of 20 μL aliquots. The resulting samples were subject to LC-MS/MS analysis using an SRM method with deuterium-labeled rucaparib (rucaparib-d_4_) as IS. The LC consisted of a Polaris C18-A column (2.1 mm × 50 mm; 3 μm, Agilent Technologies, Santa Clara, CA) and an isocratic elution with 20% acetonitrile and 0.1% formic acid in water at a flow rate of 500 μL/min. The MS/MS consisted of an AB SCIEX API4000 system and was operated with Analyst version 1.6.2 in TurboIonSpray and positive ionization mode. Rucaparib and rucaparib-d_4_ were quantified by SRMs of m/z 324.1→293.1 and m/z 331.2→300.2, respectively. The concentration range for quantitation was 5–10,000 ng/mL. Plasma concentrations below the lower limit of quantitation (5 ng/mL) were treated as “0” when calculating summary statistics.

The analysis for oral contraceptives in plasma samples was performed by PRA Health Sciences (Assen, the Netherlands), using validated LC-MS/MS methods. Plasma concentrations of ethinylestradiol and levonorgestrel were determined simultaneously. Briefly, 250 µL plasma samples with ISs (ethinylestradiol˗d_4_ for ethinylestradiol and norgestrel˗d_6_ for levonorgestrel) were processed using liquid-liquid extraction, followed by dansylation of ethinylestradiol (derivatization), and followed by a second liquid-liquid extraction. Separation between potential metabolites and interfering endogenous compounds was achieved by ultra-high performance liquid chromatography with a CSH C18 column at 40°C and gradient elution using 0.1% formic acid in water as mobile phase A and acetonitrile as mobile phase B, operating at an initial flow rate of 0.800 mL/min. A triple quadrupole mass spectrometer (Triple Quad 6500) equipped with a turbo ion spray source was used for detection in positive ion mode. Quantification is based on multiple reaction monitoring of the transitions of 530.2→171.0, 530.2→171.1, 530.2→171.2, 530.2→171.3, 530.2→171.4 for ethinylestradiol, 313.1→245.1, 313.1→245.2, 313.1→245.3 for levonorgestrel, 534.2→171.2 for ethinylestradiol˗d_4_, and 319.1→251.2 for Norgestrel˗d_6_. The concentration ranges for quantitation were 0.004–4 ng/mL for ethinylestradiol and 0.1–100 ng/mL for levonorgestrel.

## **Online Resource 2** Arithmetic mean C_min_ of rucaparib (PK analysis population)


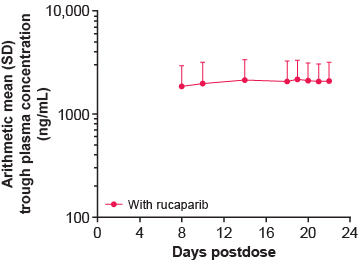


*C_min_* trough plasma concentration

Lower limit of quantitation = 5 ng/mL

Mean was not shown if more than half of the observations per time point were below quantifiable limit

## **Online Resource 3** Summary of TEAEs (safety population)

**A** Rucaparib + rosuvastatin (Arm A)

| **System Organ Class**  Preferred Term^a^ | **Maximum severity^b^** | **Prior to rucaparib (*n* = 18)** | **Rucaparib alone (*n* = 17)** | **Rucaparib + rosuvastatin (*n* = 16)** |
| --- | --- | --- | --- | --- |
| **Total patients with events, *n* (%)** | **1** | **2 (11.1)** | **5 (29.4)** | **1 (6.3)** |
|  | **2** | **0** | **5 (29.4)** | **3 (18.8)** |
|  | **3** | **0** | **1 (5.9)** | **0** |
|  | **4** | **0** | **1 (5.9)** | **0** |
| **Cardiac disorders, *n* (%)** | **2** | **0** | **1 (5.9)** | **0** |
| Sinus tachycardia | 2 | 0 | 1 (5.9) | 0 |
| **Gastrointestinal disorders, *n* (%)** | **1** | **2 (11.1)** | **3 (17.6)** | **0** |
|  | **2** | **0** | **3 (17.6)** | **1 (6.3)** |
| Abdominal distension | 1 | 0 | 1 (5.9) | 0 |
| Abdominal pain | 1 | 0 | 1 (5.9) | 0 |
|  | 2 | 0 | 1 (5.9) | 0 |
| Abdominal pain upper | 2 | 0 | 1 (5.9) | 0 |
| Diarrhea | 1 | 1 (5.6) | 2 (11.8) | 0 |
|  | 2 | 0 | 1 (5.9) | 0 |
| Dyspepsia | 1 | 0 | 1 (5.9) | 0 |
|  | 2 | 0 | 0 | 1 (6.3) |
| Vomiting | 1 | 1 (5.6) | 0 | 0 |
|  | 2 | 0 | 1 (5.9) | 0 |
| **General disorders and administration site conditions, *n* (%)** | **1** | **0** | **1 (5.9)** | **0** |
|  | **2** | **0** | **2 (11.8)** | **0** |
| Noncardiac chest pain | 2 | 0 | 1 (5.9) | 0 |
| Peripheral swelling | 1 | 0 | 1 (5.9) | 0 |
|  | 2 | 0 | 1 (5.9) | 0 |
| **Infections and infestations, *n* (%)** | **1** | **0** | **1 (5.9)** | **0** |
|  | **2** | **0** | **1 (5.9)** | **1 (6.3)** |
| Nasopharyngitis | 1 | 0 | 1 (5.9) | 0 |
| Urinary tract infection | 2 | 0 | 1 (5.9) | 1 (6.3) |
| **Investigations, *n* (%)** | **1** | **0** | **2 (11.8)** | **0** |
|  | **2** | **0** | **0** | **2 (12.5)** |
| Blood creatinine increased | 1 | 0 | 1 (5.9) | 0 |
| Body temperature increased | 2 | 0 | 0 | 1 (6.3) |
| Electrocardiogram QT prolonged | 1 | 0 | 1 (5.9) | 0 |
|  | 2 | 0 | 0 | 1 (6.3) |
| **Reproductive system and breast disorders, *n (%)*** | **1** | **0** | **0** | **1 (6.3)** |
| Vaginal hemorrhage | 1 | 0 | 0 | 1 (6.3) |
| **Respiratory, thoracic, and mediastinal disorders, *n (%)*** | **4** | **0** | **1 (5.9)** | **0** |
| Pulmonary embolism | 4 | 0 | 1 (5.9) | 0 |
| **Vascular disorders, *n (%)*** | **3** | **0** | **1 (5.9)** | **0** |
| Deep vein thrombosis | 3 | 0 | 1 (5.9) | 0 |
| *N* total number of patients, *n* number of patients with at least 1 TEAE, *TEAE* treatment-emergent adverse event  ^a^MedDRA version 22.1  ^b^According to the National Cancer Institute Common Terminology Criteria for Adverse Events version 4.03. Patients were counted under the category of their maximum severity within the preferred term | | | | |

**B** Rucaparib + oral contraceptives (Arm B)

| **System Organ Class**  Preferred Term^a^ | **Maximum severity^b^** | **Prior to rucaparib (*n* = 18)** | **Rucaparib alone (*n* = 18)** | **Rucaparib + oral contraceptives (*n* = 17)** |
| --- | --- | --- | --- | --- |
| **Total patients with events, *n* (%)** | **1** | **1 (5.6)** | **8 (44.4)** | **2 (11.8)** |
|  | **2** | **1 (5.6)** | **1 (5.6)** | **3 (17.6)** |
|  | **3** | **0** | **2 (11.1)** | **1 (5.9)** |
| **Blood and lymphatic system disorders, *n* (%)** | **1** | **0** | **1 (5.6)** | **0** |
|  | **2** | **0** | **0** | **2 (11.8)** |
| Anemia | 1 | 0 | 1 (5.6) | 0 |
|  | 2 | 0 | 0 | 1 (5.9) |
| Thrombocytopenia | 2 | 0 | 0 | 1 (5.9) |
| **Gastrointestinal disorders, *n* (%)** | **1** | **1 (5.6)** | **5 (27.8)** | **2 (11.8)** |
|  | **2** | **1 (5.6)** | **0** | **0** |
|  | **3** | **0** | **1 (5.6)** | **0** |
| Abdominal discomfort | 1 | 0 | 1 (5.6) | 0 |
| Abdominal pain | 1 | 0 | 1 (5.6) | 1 (5.9) |
| Ascites | 3 | 0 | 1 (5.6) | 0 |
| Diarrhea | 1 | 1 (5.6) | 0 | 0 |
| Nausea | 1 | 0 | 1 (5.6) | 1 (5.9) |
| Vomiting | 1 | 0 | 2 (11.1) | 1 (5.9) |
|  | 2 | 1 (5.6) | 0 | 0 |
| **General disorders and administration site conditions, *n* (%)** | **1** | **0** | **1 (5.6)** | **0** |
| Peripheral swelling | 1 | 0 | 1 (5.6) | 0 |
| **Infections and infestations, *n* (%)** | **1** | **0** | **1 (5.6)** | **0** |
| Pharyngitis bacterial | 1 | 0 | 1 (5.6) | 0 |
| **Injury, poisoning, and procedural complications, *n* (%)** | **1** | **0** | **1 (5.6)** | **0** |
| Accident | 1 | 0 | 1 (5.6) | 0 |
| Arthropod bite | 1 | 0 | 1 (5.6) | 0 |
| **Investigations, *n* (%)** | **2** | **0** | **0** | **1 (5.9)** |
|  | **3** | **0** | **1 (5.6)** | **0** |
| Alanine aminotransferase increased | 2 | 0 | 0 | 1 (5.9) |
|  | 3 | 0 | 1 (5.6) | 0 |
| Aspartate aminotransferase increased | 3 | 0 | 1 (5.6) | 0 |
| **Musculoskeletal and connective tissue disorders, *n (%)*** | **1** | **0** | **1 (5.6)** | **0** |
| Musculoskeletal pain | 1 | 0 | 1 (5.6) | 0 |
| **Nervous system disorders, *n (%)*** | **1** | **0** | **3 (16.7)** | **0** |
|  | **3** | **0** | **0** | **1 (5.9)** |
| Cerebrovascular accident | 3 | 0 | 0 | 1 (5.9) |
| Dysgeusia | 1 | 0 | 2 (11.1) | 0 |
| Headache | 1 | 0 | 1 (5.6) | 0 |
| Somnolence | 1 | 0 | 1 (5.6) | 0 |
| Taste disorder | 1 | 0 | 1 (5.6) | 0 |
| **Psychiatric disorders, *n (%)*** | **1** | **0** | **1 (5.6)** | **0** |
| Apathy | 1 | 0 | 1 (5.6) | 0 |
| **Renal and urinary disorders, *n (%)*** | **3** | **0** | **1 (5.6)** | **0** |
| Acute kidney injury | 3 | 0 | 1 (5.6) | 0 |
| **Skin and subcutaneous tissue disorders, *n (%)*** | **1** | **0** | **1 (5.6)** | **1 (5.9)** |
|  | **2** | **0** | **1 (5.6)** | **0** |
| Erythema | 1 | 0 | 0 | 1 (5.9) |
| Onycholysis | 1 | 0 | 1 (5.6) | 0 |
| Skin hemorrhage | 2 | 0 | 1 (5.6) | 0 |
| *N* total number of patients, *n* number of patients with at least 1 TEAE, *TEAE* treatment-emergent adverse event  ^a^MedDRA version 22.1  ^b^According to the National Cancer Institute Common Terminology Criteria for Adverse Events version 4.03. Patients were counted under the category of their maximum severity within the preferred term | | | | |
